# Successful use of eculizumab to treat atypical hemolytic uremic syndrome in patients with inflammatory bowel disease

**DOI:** 10.1186/s12959-019-0207-7

**Published:** 2019-09-09

**Authors:** Ramy M. Hanna, Noah Merin, Richard M. Burwick, Lama Abdelnour, Umut Selamet, Beshoy Yanny, Patrick Bui, Mary Fouad, Ira Kurtz

**Affiliations:** 1 Department of Medicine, Division of Nephrology, UCLA David Geffen School of Medicine, Room 7-155, Factor Bldg. 700 Tiverton Ave, Los Angeles, CA 90095 USA; 2 Department of Medicine, Division of Nephrology, University of California Irvine School of Medicine, Irvine CA, USA; 3 Department of Medicine, Division of Hematology Oncology, Cedars Sinai Medical Center, Los Angeles, USA; 4 Department of Obstetrics and Gynecology, Division of Maternal Fetal Medicine, Cedars Sinai Medical Center, Los Angeles, USA; 5 Department of Medicine, Division of Digestive Diseases, UCLA David Geffen School of Medicine, Los Angeles, USA; 6 Department of Medicine, UCLA David Geffen School of Medicine, Los Angeles, USA; 7 UCLA Brain Research Institute, Los Angeles, USA

**Keywords:** Atypical hemolytic uremic syndrome, Complement, Complement blockade, Thrombotic Microangiopathy, Inflammatory bowel disease, Crohn’s colitis/Crohn’s disease, Ulcerative colitis

## Abstract

**Background:**

Atypical hemolytic uremic syndrome is a rare group of disorders that have in common underlying complement amplifying conditions. These conditions can accelerate complement activation that results in a positive feedback cycle. The known triggers for complement activation can be diverse and include, infection, autoimmune disease, and malignancy. Recent reports suggest that certain autoimmune and rheumatological triggers of complement activation may result in atypical hemolytic uremic syndrome that does not resolve despite treating the underlying disorder. Specifically, patients with systemic lupus erythematosus and microangiopathic hemolysis may not respond to treatment of their underlying rheumatological trigger but responded to complement blockade.

**Case presentations:**

We report two patients with inflammatory bowel disease complicated by development of atypical hemolytic uremic syndrome. In both cases, patients were on treatment for inflammatory bowel disease, that was not well controlled/flaring at the time. The first patient is a male who developed Crohn’s disease and microangiopathic hemolysis at age 5 and was treated with eculizumab successfully. Discontinuation of the medication led to multiple relapses, and the patient currently is being treated with eculizumab and has normal hematological and stable renal parameters. The second patient is a 49-year-old female with Ulcerative Colitis treated with 6-Mercaptopurine. She developed acute kidney injury and microangiopathic hemolysis. Prompt diagnosis and treatment with eculizumab resulted in the recovery of kidney injury along with a complete hematological response.

**Conclusions:**

These two cases are the fifth and sixth patients to be published in the literature with atypical hemolytic uremic syndrome and inflammatory bowel disease treated with complement blockade. This confirms that C5 complement blockade is effective in treating complement mediated thrombotic microangiopathy/atypical hemolytic uremic syndrome when it is triggered in patients with inflammatory bowel disease.

## Key points

1. IBD can trigger aHUS, and in both cases, we report patients received eculizumab and other biologic agents for IBD concurrently.
2. We report the 5th and 6th cases of complement mediated TMA/aHUS in IBD patients (1 with UC and 1 with CD), with successful resolution of aHUS with complement blockade.
3. We report prevention of relapse with ongoing complement blockade in one case.

## Background

Atypical Hemolytic Uremic Syndrome (aHUS), is a syndrome resulting from complement activation. The causes of complement activation are numerous, and the genetics are diverse. In affected patients, a latent susceptibility becomes unmasked by a complement amplifying condition resulting in Thrombotic Microangiopathy (TMA) [1]. New research is revealing complement biology as having a role in microangiopathic hemolysis and endothelial dysfunction in many rheumatological diseases [1]. Recent publications by Park et al. disclose that ongoing microangiopathic hemolysis stemming from rheumatological conditions like systemic lupus erythematosus can be treated with eculizumab when more traditional immunosuppression has failed [2, 3]. Microangiopathic hemolysis due to Antiphospholipid antibody syndrome and catastrophic antiphospholipid antibody syndrome (CAPS) variant has also been successfully treated with complement blockade [4].

The final common pathway of a positive feedback complement activation loop is the etiologic feature of aHUS, despite the various triggers [1]. In most cases, a strong genetic susceptibility is unmasked by the presence of a strong temporary, or a milder but lingering complement amplifying condition [1]. Indeed, some cases of aHUS can be self-limiting (due to hypertension, post-partum aHUS), and others can be due to slow ongoing complement activation (C3 glomerulopathy, and chronic hypertension due to occult TMA) [1].

Inflammatory bowel disease (IBD), resulting in Crohn’s Disease (CD) and Ulcerative Colitis (UC) as clinical phenotypes, is one such group of complement amplifying conditions [5]. As with other autoimmune diseases, first-line treatment has always focused on suppression of the immune system to eliminate inflammation and clinical symptoms. In the current literature, there are three published case reports and one abstract detailing patients with IBD and aHUS treated with eculizumab [6–10].

We report two additional cases of patients with IBD and aHUS treated with eculizumab. Patient 1 is a 19-year-old male with CD who was treated with eculizumab at age 5 and had multiple relapses off the drug. He was maintained on eculizumab with near-normal renal and hematological parameters for 14 years. Patient 2 is a 49-year-old female with UC who developed severe aHUS and was promptly treated with eculizumab resulting in normalization of renal and hematological parameters.

## Case presentations

### Patient 1

The first patient is a 19-year-old Caucasian male who presented for the continuation of complement blockade therapy with eculizumab. He was diagnosed with CD at age 5 and started on per rectum steroid therapy with budesonide and mesalamine therapy. He did require continuous infusions of adalimumab then infliximab for control of his disease. During the initial episode of colitis/CD in 2005, he was noted to have thrombocytopenia, hemolytic anemia, schistocytes with elevations in lactate dehydrogenase. The ADAMTS13 level was not available early on in the time of his diagnosis. Though Shiga toxin was not found in stool, vitamin B12 levels was 781 nanogram (ng)/mL, red blood cell (RBC) folate levels were within normal limits 981 nanomoles/L excluding other known TMA causes at that time. Rheumatolgoic serologies like anti nuclear antibody (ANA), anti double stranded DNA (dsDNA), anti ribonucleic protein smith (RNP-smith), anti cardiolipin (anti CL), dilute russell viper venom time assay (DRVVT), and anti phospholipid antibody (anti APL Ab) studies were all negative during initial workup.

As the scientific understanding of aHUS developed, it was decided that eculizumab would be initiated on an experimental basis with institutional review board approval (in a health system in Florida). He underwent a several-month course of eculizumab with clinical response. Following discontinuation of the medication, the patient suffered a relapse a few months later. Therapy was reinitiated and was subsequently weaned off successfully. Complement testing found a high level of C5 convertase function with high soluble membrane attack complex that improved with eculizumab use.

In April 2010 the patient had a severe flare of his underlying CD despite ongoing use of adalimumab, his disease remained poorly controlled clinically. He had a concomitant serum creatinine elevation peaking at 3.13 mg/dL in 04/2010 (baseline of 0.57 mg/dL 08/2009) with a drop in hemoglobin from 12.9 g/L baseline in 08/2009 to 7.6 g/L in 03/2010. The total bilirubin concentration was not elevated but increased from a baseline of 0.1 (mg/dL) in 03/2010 to 0.8 (mg/dL) in 04/2010 during the flare. Lactate dehydrogenase (LDH) was significantly elevated at 2223 Units/L in 4/2010 and decreased after that. The platelets had decreased by ~ 50% from 366,000/uL at baseline in 02/2010 to 187,000/uL in 4/2010. ADAMTS 13 activity level was within normal limits at 44% ruling out ADAMTS13 deficiency and thrombotic thrombocytopenic purpura (TTP). Prothrombin time (PT) was 11 s with an International Normalized Ratio (INR) was 1, and activated partial thromboplastin time (aPTT) was 39 s both within normal. Fibrinogen levels were high at 471 mg/dL, and a D-dimer was high at 6.17 microgram/mL as expected in an inflammatory condition. Absolute reticulocyte count was in the upper limit of normal / slightly elevated at 2.92%.

Since the patient had an established aHUS diagnosis, eculizumab was promptly reintroduced at 900 mg (infusion dose) each week with normalization of the platelet count and LDH levels, and improvement of the serum Cr to 1 mg/dL. The patient received eculizumab (1200 mg infusions) every two weeks for 8.5 years continually after this aHUS relapse. The patient had already received appropriate meningococcal vaccinations from prior courses and was aware of the risks and benefits of pharmacological complement blockade. He has not had any issues with meningitis or other infections linked to eculizumab. Clinically, his IBD symptoms slowly improved with use of ongoing adalimumab and steroid therapy for CD/IBD along with eculizumab use for aHUS.

**Fig. 1:**
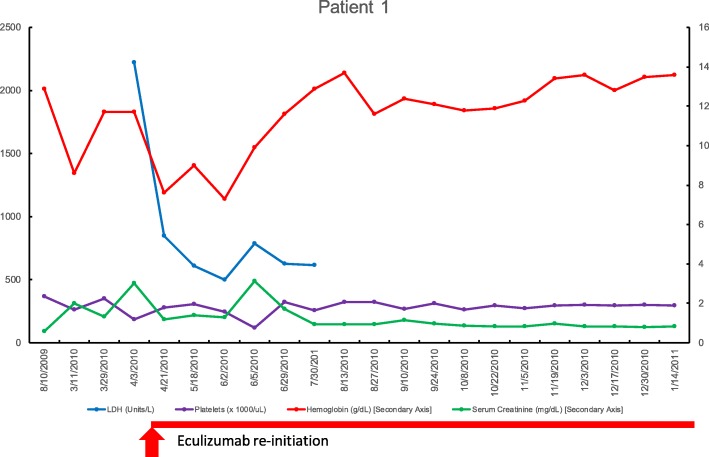
Laboratory tests for patient one as a function of time. Abbreviations: dL, deciliter; g, gram; L, liter; LDH, lactate dehydrogenase; mg, milligram; uL, microliter

The effect of this regimen was the maintenance on his aHUS was the maintenance of normal hematological parameters and renal function with current serum Cr of 1 mg/dL, hemoglobin of 10–12 g/L, and LDH within normal limits. The patient has agreed to continue lifelong eculizumab in this case given his two prior relapses. No antibodies against complement factors were found, and no mutations were found. Only 60% of patients with aHUS have detectable mutations, and in some cases, the mutations can be present but are currently unknown [11]. Figure 1 details laboratory findings in patient 1 and his course during his last flare in 2010.

### Patient 2

The second patient is a 49-year-old Caucasian female with a history of UC treated with six mercaptopurine (6MP), and steroids per rectum [budesonide]. She developed a flare of ulcerative colitis symptoms, along with increased platelet count from 322,000 platelets/uL (09/2018) to 622,000 platelets/uL (10/2018). This was a reactive thrombocytosis that occurred initially, and was clinically concomitant with a flare despite treatment with budesonide and 6MP.Her serum Cr at baseline was 0.51 mg/dL, and her hemoglobin was 13.1 g/L. She presented to the emergency department one month after the start of this flare with weakness and was found to have a decreased hemoglobin concentration of 4.3 g/L, serum Cr of 3.2 mg/dL, LDH of 1000 units/L, and schistocytes on the peripheral smear. Her platelets had droped from baseline of 322,000 platelets/ul and more recent peak of 622,000 platelets/ul to 83,000 platelets/ul; this level dropped further to 43,000 plateltes/ul at a nadir 11/17/2018. These findings and the hemolysis suggested thrombotic microangiopathy like aHUS as the likely diagnosis over 6-mercaptopurine bone marrow toxicity which was another possibility. Vitamin B12 level was normal at 769 ng/mL, serum folate level was also normal at 12.9 ng/mL. PT was 13 s with an INR of 1.3, aPTT was slightly elevated at 39 s. Fibrinogen levels were high normal range at 313 mg/dL, with an elevated D-dimer 3.28 microgram/mL. Rheumatologic serologies (ANA, anti dsDNA, anti RNP sm, anti CL, DRVVT, APL Ab) were all negative. Reticulocyte counts were obtained at 2% (normal), and one result was 3% which is slightly elevated, and not expected in bone marrow toxicity.

The finding of an ADAMTS13 level of 80.8% suggested that the cause of TMA was not TTP. The patient was transferred back to a quaternary care center, and then eculizumab was started after meningococcal vaccination. The patient agreed to the risks and benefits of complement blockade in the absence of waiting the requisite two weeks for development of meningococcal antibody titers after immunization, and antibiotic prophylaxis with penicillin-based antibiotics was provided.

**Fig. 2:**
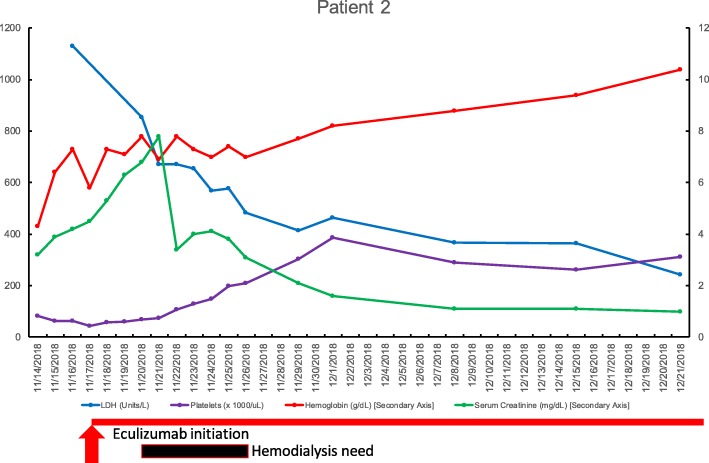
Laboratory results for patient two as a function of time. Abbreviations: dL, deciliter; g, gram; L, liter; LDH, lactate dehydrogenase; mg, milligram; uL, microliter

The serum Cr, platelets, hemoglobin, LDH all improved quickly and normalized (Fig. 2). The patient required hemodialysis for ~ 1 week and was weaned off. Eculizumab was administered at a given dose of 900 mg weekly infusions. The patient was discharged on 1200 mg infusions of eculizumab every other week with normal lab parameters. She is currently undergoing genetic testing at the University of Iowa laboratory to look for autoantibodies and genetic mutations. The response to eculizumab and the clinical course are typical for aHUS and diagnostic.

## Discussion and conclusions

We report a series of two patients with two variants of IBD presented with a very rare association with aHUS. The pathophysiology of aberrant complement activation does not require a specific trigger, and like rheumatological diseases, gastroenterological autoimmune disease like IBD can also trigger complement activation in susceptible individuals [1]. Interestingly, both patients had poorly controlled IBD at the time of aHUS diagnosis. The patients’ IBD (CD case 1, UC case 2), was eventually well controlled with intensive IBD therapy, alongside complement blockade for aHUS.

The lack of finding a complement mutation in patient 1 who had a relapsing course is suggestive of either: 1) a new complement mutation that has not been previously reported; 2) background gene effect. The two known classes of complement mutations are complement factor mutations resulting in auto-activation or increased activation of complement (factor B, factor I), and loss of function mutations resulting in decreased ability of self-cells to disarm complement proteins (like membrane cofactor protein, etc.) [11]. Other complement proteins interact with the complement cascade that when mutated can also affect complement function. For example, vitronectin is the newest genetic defect described that has been linked to aHUS [11, 12].

In addition to four other published case, we report the fifth and sixth cases of IBD associated aHUS successfully treated with eculizumab. Table 1 summarizes previous case reports in the literature and our current cases that highlight the infrequent association between IBD (CD and UC) and aHUS [6–10].

**Table 1:** aHUS Cases Associated with Inflammatory Bowel Disease

Reference #	n	Pt age	Pt gender	UC/CD	Creatinine improvement (Y/N)	TMA resolution (Y/N)	Ab/Mutation found?
Case Reports							
Beers [6] [abstract]	1	19	F	IBD**	Y	Y	CFH Auto-Ab & HZ CF1/CFH3 Del
Webb [7]	1	16	M	UC	Y	Y	HZ MTHFR mutation***
Viada Bris [8, 9]*	1	15	F	UC	NT	NT	HZ CFH, HZ MCP
Green H [10]	1	27	F	UC	Y	Y	CFH Auto-Ab
Hanna et.al. Case 1	(1/2)	19	M	CD	Y	Y	No clear mutation found****
Hanna et.al. Case 2	(2/2)	49	F	UC	Y	Y	Testing not completed

*Ab* antibody, *CD* Crohn’s disease, *CF1* complement factor 1, *CF3* complement factor 3, *CFH* complement factor H, *HZ* heterozygous, *MCP* membrane cofactor protein, *MTHFR* methylenetetrahydrofolate reductase gene/protein product, *n* number, *N* no, *NT* not treated, *Pt* patient, *UC* ulcerative colitis, *Y* yes. *8, 9 describe same case. ** IBD is only stated, it is not otherwise specified whether UC/CD.***Heterozygous MTHFR mutations are not generally thought to cause aHUS, therefore a known causative mutation was not found in Webb et.al. [7]. ****Early on in Case 1 a borderline positive C3 nephritic factor antibody was found and complement testing revealed a high level of soluble membrane attack complex

## Data Availability

Not applicable, no data.

## Abbreviations

6MP: Six mercaptopurine
ADAMTS13: A Disintegrin and Metalloproteinase Thrombospondin Motif # 13, member 1
aHUS: Atypical Hemolytic Uremic Syndrome
ANA: Anti Nuclear Antibody
anti APL Ab: Anti Phospholipid Antibody
anti CL: Anti Cardiolipin
aPTT: Activated Partial Thromboplastin Time
CAPS: Catastrophic Antiphospholipid Antibody Syndrome
CD: Crohn’s Disease
Cr: Creatinine
dL: Deciliter
DRVVT: Dilute Russell Viper Venom Time Assay
dsDNA: Anti Double Stranded DNA
IBD: Inflammatory bowel disease
INR: International Normalized Ratio
L: Liter
LDH: Lactate Dehydrogenase
mg: Milligram
microliter: uL
mL: Milliliter
ng: Nanogram
PT: Prothrombin Time
RBC: Red blood cell
RNP-smith,: Anti Ribonucleic protein Smith
TMA: Thrombotic Microangiopathy
TTP.: Thrombotic Thrombocytopenic Purpura
UC: Ulcerative Colitis
